# Structural Studies of an Anti-Inflammatory Lectin from *Canavalia boliviana* Seeds in Complex with Dimannosides

**DOI:** 10.1371/journal.pone.0097015

**Published:** 2014-05-27

**Authors:** Gustavo Arruda Bezerra, Roland Viertlmayr, Tales Rocha Moura, Plínio Delatorre, Bruno Anderson Matias Rocha, Kyria Santiago do Nascimento, Jozi Godoy Figueiredo, Ingrid Gonçalves Bezerra, Cicero Silvano Teixeira, Rafael Conceição Simões, Celso Shiniti Nagano, Nylane Maria Nunes de Alencar, Karl Gruber, Benildo Sousa Cavada

**Affiliations:** 1 Institute of Molecular Biosciences, University of Graz, Graz, Austria; 2 Department of Biochemistry and Molecular Biology, Federal University of Ceará, Fortaleza, Brazil; 3 Department of Molecular Biology, Federal University of Paraíba, João Pessoa, Brazil; 4 Department of Physiology and Pharmacology, Federal University of Ceará, Fortaleza, Brazil; 5 Department of Fishing Engineering, Federal University of Ceará, Fortaleza, Brazil; NCI-Frederick, United States of America

## Abstract

Plant lectins, especially those purified from species of the Leguminosae family, represent the best-studied group of carbohydrate-binding proteins. Lectins purified from seeds of the *Diocleinae* subtribe exhibit a high degree of sequence identity notwithstanding that they show very distinct biological activities. Two main factors have been related to this feature: variance in key residues influencing the carbohydrate-binding site geometry and differences in the pH-dependent oligomeric state profile. In this work, we have isolated a lectin from *Canavalia boliviana* (Cbol) and solved its x-ray crystal structure in the unbound form and in complex with the carbohydrates Man(α1-3)Man(α1-O)Me, Man(α1-4)Man(α1-O)Me and 5-bromo-4-chloro-3-indolyl-α-D-mannose. We evaluated its oligomerization profile at different pH values using Small Angle X-ray Scattering and compared it to that of Concanavalin A. Based on predicted pKa-shifts of amino acids in the subunit interfaces we devised a model for the dimer-tetramer equilibrium phenomena of these proteins. Additionally, we demonstrated Cbol anti-inflammatory properties and further characterized them using *in vivo* and *in vitro* models.

## Introduction

Lectins are carbohydrate binding proteins unrelated to immunoglobulins that display no enzymatic activity towards the recognized sugars [1]. They are responsible for deciphering the glyco codes [2] playing a central role in various biological events such as infections and cell communication and growth [3]. The above cited features elect these proteins as useful tools in bioscience and biomedicine [4]. Plant lectins are the most well studied group, although their functional role is not fully understood yet. The carbohydrate recognition process by a protein is a complex problem [5] involving various types of forces and interactions. Legume lectins are widely used as model systems for studying these interactions because they are relatively easy to purify and they cover a wide range of carbohydrate specificities [6].

Lectins purified from seeds of the *Diocleinae* subtribe exhibit a high degree of sequence identity notwithstanding that they show very distinct biological activities [7]; [8]; [9]; [10]; [11]. This remarkable feature can be explained by several factors. It has been reported that the relative position of the carbohydrate-binding site and pH-dependent dimer-tetramer equilibrium contribute to these differences [12]; [13]; [14]. The substitution of one amino acid residue related to the carbohydrate-binding site has been held responsible for a more open carbohydrate-binding site in the lectin from *Canavalia brasiliensis* compared to ConA (*Canavalia ensiformis* lectin) resulting in distinct activities [15]; [16]. Minor changes in the amino acid composition are also responsible for the different affinities and biological activities of the *Canavalia maritima* lectin [17]; [18]; [19].

Inflammatory reactions are marked by neutrophil migration from the blood into affected tissues. This is a complex and multi-mediated process that results from the release of inflammatory mediators and involves proteins with lectin domains, the selectins [20]. These proteins, which are present both in neutrophil and endothelial cells, interact with their respective carbohydrate ligands mediating the process called rolling. New anti-inflammatory drugs are targeted to interrupt or inhibit the neutrophil migration [21]. Since lectins have the property to bind carbohydrates their ability to antagonize, *in vivo*, neutrophil migration induced by inflammatory stimuli is well established [22]; [23].

In the present study, we used mass spectrometry to determine the amino acid sequence of a lectin from *Canavalia boliviana* seeds and solved its crystal structure in complex with Xman and the dimannosides Man(α1-3)Man(α1-O)Me and Man(α1-4)Man(α1-O)Me. In addition we evaluated its anti-inflammatory properties using *in vivo* and *in vitro* models.

## Materials and Methods

### Purification, Digestion and Sequencing

The protein was purified according to Moura *et al*., 2009, [24]. Purified Cbol was submitted to an SDS-PAGE and mass spectrometry analysis. The average molecular mass of Cbol was determined by electrospray ionization using a hybrid quadrupole/ion mobility separator/orthogonal acceleration-time of flight mass spectrometer (Synapt HDMS system-Waters Corp., Milford, USA). The protein suspension (10 ρmol/µL) was infused into the system at a flow rate of 1 µL/min. The capillary voltage and the cone voltage were set at 3 kV and 40 V, respectively. The source temperature was maintained at 100°C and nitrogen was used as a drying gas (flow rate of 150 L/h). The acquisition of data was performed by Mass Lynx 4.0 software and the multiply charge spectra were deconvoluted using maximum entropy techniques [25]. Protein digestion was carried out as previously described by Shevchenko and co-workers (2006) [26]. For this, the protein was submitted to SDS-PAGE and the Coomasie stained bands were excised and then bleached in a solution of 50 mM ammonium bicarbonate in 50% acetonitrile. The bands were then dehydrated in 100% acetonitrile and dried in a speedvac (LabConco). The gels were rehydrated with the following enzyme solutions: 50 mM ammonium bicarbonate containing trypsin (Promega, Madison, WI, USA), chymotrypsin (Sigma-Aldrich) and 10 mM HCl containing pepsin (Sigma-Aldrich) (1∶50 w/w; enzyme∶substrate ratio) at 37°C overnight. The peptides were then extracted in a solution of 50% acetonitrile with 5% formic acid and then concentrated in speedvac. The peptides were separated on a C18 chromatography column (75 µm × 100 mm) using a nanoAcquity system and eluted with an acetonitrile gradient (10%–85%), containing 0.1% formic acid. The liquid chromatography was connected to a nanoelectrospray mass spectrometer source (SYNAPT HDMS system-Waters Corp., Milford, USA). The mass spectrometer was operated in positive mode, using a source temperature of 80°C and capillary voltage at 3.5 kV. The instrument was calibrated with the doubly protonated ion of glucofibrinopeptide B *m/z* 785.84. The LC-MS/MS experiment was used according to DDA (Data Dependent Acquisition) function selecting for the experiments of MS/MS double or triple charged precursor ions, which were fragmented by collision induced dissociation (CID) using a ramp collision energy which varied according to the charge state of precursor ion. The data were processed and analyzed using the ProteinLynx Global Server (Waters), using ‘peptide fragmentation pattern’ as the search parameter. Some peptide sequences were obtained by *de novo* manual sequencing followed by manual interpretation of CID spectra. The sequence obtained was then analyzed by local and multiple alignments performed with the aid of BLAST [27] and CLUSTALW [28], respectively.

### Crystallization, Structure Determination and Refinement of the Unbound Cbol

An Oryx8 Protein Crystallization Robot from Douglas Instruments was used for setting up the initial crystallization trials by the sitting-drop vapour diffusion method. The drops had a total volume of 1.4 µL consisting of 0.7 µL Cbol at 5 mg/mL + 0.7 µL of reservoir solution. The system was then equilibrated against 100 µL of reservoir solution, at 20°C. Cbol crystals grew in condition 22 of the Morpheus screen (Molecular Dimensions): 0.09 M halogens (NaF, NaBr, NaI), 0.1 M trizma and bicine at a pH 8.5, 30% ethylene glycol and polyethylene glycol 8000. A 2.9 Å data set was collected at beamline BM14 at the European Synchrotron Radiation Facility (ESRF), in Grenoble, France. The data were processed using Mosflm [29] and Scala to 3.4 Å [30]. The structure was solved by molecular replacement using the software PHASER [31] with the structure of Cbol in complex with Xman (see below) as search template. The model was refined with PHENIX [32] and COOT [33] to an R_cryst_ and R_free_ of 0.193 and 0.263, respectively, at 3.4 Å. The model was validated using MOLPROBITY [34] and deposited to the Protein Data Bank (PDB code: 4K20). Details of the data collection, processing and structure refinement are summarized in **Table 1**.

**Table 1:** Data collection and refinement statistics.

	Cbol	Cbol:Xman	Cbol:M13M	Cbol:M14M
**Data collection**				
Beamline	BM14 ESRF	LNLS MX2	SLS PX3	SLS PX3
Wavelength (Å)	0.9790	1.4558	0.9999	0.9999
	*a* = 71.63 Å	*a* = 64.97 Å	*a* = 139.88 Å	*a* = 107.81 Å
Unit cell	*b* = 71.63 Å	*b* = 66.59 Å	*b* = 95.66 Å	*b* = 107.81 Å
	*c* = 167.79 Å	*c* = 108.77 Å	*c* = 94.61 Å	*c* = 157.9 Å
			β = 132.29°	
Space group	*P*3_1_2	*I222*	*C*2	*P*4_1_2_1_2_1_
Resolution range (Å)*	62–3.4 (3.58–3.4)	28–1.6 (1.69–1.60)	41–2.5 (2.64–2.50)	41–2.3 (2.42–2.30)
Completeness (%)	100.0 (100.0)	95.1 (73)	99.9 (100.0)	100.0 (100.0)
Redundancy	10.2 (10.3)	6.4 (3.2)	5.6 (5.6)	29.2 (29.7)
R_sym_	0.130 (0.497)	0.068 (0.207)	0.139 (0.678)	0.107 (0.259)
<I/σ(I)>	13.2 (4.7)	17.4 (4.2)	9.5 (2.5)	26.4 (13.3)
Unique reflections	7344	29896	31961	42106
**Refinement**				
R/R_free_	0.193/0.263	0.146/0.192	0.204/0.228	0.161/0.222
r.m.s.-deviations				
bond length (Å)	0.009	0.010	0.009	0.004
bond angle (°)	1.147	1.314	1.032	0.882
Number of atoms				
protein	3628	1873	7228	7225
metal ions	4	2	17	19
carbohydrate	-	23	96	96
water	27	187	188	539
B-factors (Å^2^)				
protein	83.79	21.9	32.07	25.63
metal ions	75.04	18.5	46.52	30.0
carbohydrate	-	18.8	36.38	43.0
water	41.66	36.2	29.8	30.3

*Values for the highest resolution shell are given in parentheses.

### Crystallization, Structure Determination and Refinement of Cbol in Complex with Xman

The crystallization procedure, data collection and processing of Cbol in complex with Xman were previously described in detail [24]. The diffraction data set was indexed and integrated using MOSFLM [29] and the intensities reduced using SCALA [30]. The phase problem was solved by molecular replacement method using the program BALBES [35]. The structure of ConA (PDB code: 1NLS, [36]) was used as search template. The model was refined with PHENIX [32] and COOT [33] to an R_cryst_ and R_free_ of 0.146 and 0.192, respectively, at 1.6 Å. The model was validated using MOLPROBITY [34] and deposited to the Protein Data Bank (PDB code: 4K21). Details of the data collection, processing and structure refinement are summarized in **Table 1**.

### Crystallization, Structure Determination and Refinement of Cbol in Complex with Man(α1-3)Man(α1-O)Me and Man(α1-4)Man(α1-O)Me

An Oryx8 Protein Crystallization Robot from Douglas Instruments was used for setting up the initial crystallization trials by the sitting-drop vapour diffusion method. 4 mM of the dimannosides Man(α1-3)Man(α1-O)Me (Calbiochem) and Man(α1-4)Man(α1-O)Me (Sigma-Aldrich) were added to Cbol sample at 5 mg/mL (0.2 mM), corresponding to a 20 fold excess. The drops had a total volume of 1.4 µL consisting of 0.7 µL Cbol at 5 mg/mL + 0.7 µL of reservoir solution. The system was then equilibrated against 100 µL of reservoir solution, at 20°C. Crystals of Cbol + M13M and Cbol + M14M were grown in condition 12 of Crystal Screen II (Hampton Research): 0.1 M cadmium chloride hydrate, 0.1 M sodium acetate trihydrate pH 4.6 and 30% (v/v) polyethylene glycol 400. Optimization trials were unsuccessful and the best diffracting crystals grew in the original condition.

Data set were collected at the beamline PX3 at the Swiss Light Source (SLS), in Villigen, Switzerland. The data were processed using Mosflm [29] and Scala [30]. The structures were solved by molecular replacement using the software PHASER [31] and Cbol in complex with Xman as search template. Cbol:M13M model was refined with PHENIX [32] and COOT [33] to a R_cryst_ and R_free_ of 0.204 and 0.228, respectively, at 2.5 Å. Processing and refinement for Cbol:M14M was realized as described above, resulting in a model with R_cryst_ and R_free_ of 0.161 and 0.222, respectively, at 2.3 Å. Cbol:M13M and Cbol:M14M models were validated using MOLPROBITY [34] and deposited to the Protein Data Bank (PDB codes: 4K1Y and 4K1Z, respectively). Details of the data collection, processing and structure refinement are summarized in **Table 1**.

### Measurement of SAXS Data

The data collection for the Small Angle X-ray Scattering (SAXS) studies of the pH-dependent oligomerization of *Canavalia boliviana* lectin (Cbol) was performed at the European Molecular Biology Laboratory (EMBL) in Hamburg, Germany. Beamline X33 was equipped with a 2D Photon counting Pilatus 1M-W pixel x-ray detector and operated at a wavelength of 0.15 nm (1.5 Å). The distance between sample and detector was 2.7 m [37]; [38]. Protein samples of Cbol and Concanavalin A (ConA) were measured at three different concentrations (1, 5 and 10 mg/ml) in different buffers depending on the desired pH. The buffer spectrum ranged between pH 3 and pH 9, using prepared stocks from the JBS Solubility Kit (Jena Bioscience, CO-310, [39]). Bovine serum albumin (BSA) at a concentration of 4.4 mg/mL was used as a size standard resulting in a forward scattering intensity (I(0)) value of 113.70 corresponding to a molecular weight of 66 kDa. Data analysis was performed using the program PRIMUS with which the scattering of the buffer was subtracted as background from the protein measurements [40]. The radius of gyration (R_G_) and the forward scattering intensity (I(0)) were estimated using the Guinier approximation [41]. Based on the I(0) values of the scattering curves measured at low, middle and high protein concentration an average molecular weight for each pH value was calculated.

### Animals and Study Design

Wistar rats (*Rattus norvergicus*) (180–220 g) were housed in appropriate cages at 25±2°C under a 12/12 h light/dark cycle, and food and water were supplied ad libitum. Experiments were carried out according to the Guide for the Care and Use of Laboratory Animals of the U.S. Department of Health and Human Services (NIH publication no. 85–23, revised 1985) and approved by the Institutional Animal Care and Use Committee of the Federal University of Ceará (UFC) (Protocol No. 57/2009), Fortaleza, Brazil.

In the experiments listed below, animals were randomized into treatment groups. Four experiments used 108 mice using six animals for treatment, varying between 4 or 5 treatment groups. All the experiments were conducted in the light phase.

All mice were submitted to physical euthanasia by cervical dislocation method under anesthesia (Ketamine 75–100 mg/kg IP) intended to be quick and painless.

### Peritonitis Model

For the determination of neutrophil migration to peritoneal cavity, CboL was administered i.v. 30 min before (0.5; 1 and 5 mg/Kg) the administration of inflammatory stimuli by intraperitoneal injection of carrageenan at 500 µg/cavity or sterile saline (0.9% w/v) (4 treatment groups). Peritoneal cavity was washed with 10 ml of saline containing 5 IU/ml heparin. The peritoneal fluid was recovered and total and differential leukocyte counts were performed. Results were expressed as means ± S.E.M. of the number of cells×10^3^/ml of peritoneal fluid [42].

### Effect of Thermal Denaturation and Glucose on Neutrophil Migration

1 ml of sterile saline solutions containing CboL (1 mg/Kg) alone, CboL (1 mg/Kg) with 0.5 M of Glucose, or Glucose (0.5 M) alone were injected i.p. into the animals. All solutions were incubated for 30 min at 37°C. Total and differential cell leukocyte counts were determined 4 h after the administration of inflammatory stimuli by intraperitoneal injection of carrageenan at 500 µg/cavity or sterile saline (0.9% w/v), resulting 5 treatment groups with positive control (carrageenan).

To investigate the importance of native structure in the anti-inflammatory effect of CboL, 1 ml of sterile saline solution containing CboL (1 mg/Kg), or CboL (1 mg/Kg) previously heat-treated for 30 min in boiling water was injected i.v. The effects were evaluated 4 h after the injection of inflammatory stimuli and compared to the saline-treated control group (4 treatment groups).

### Paw Edema

Paw edema was induced by s.c. injection of carrageenan into the right hind paw of rats at 0.5 µg/paw (0.1 ml), 30 min after i.v. treatment of animals with CboL (1 mg/Kg; 0.1 ml). Positive controls received carrageenan s.c., and negative controls the same volume of saline s.c. The binding between lectin and sugar was used to determine the involvement of sugar residues in the lectin effect. For this, animals were treated i.v. with a solution containing the most active dose of the lectin combined with 0.5 M of its ligand (Glucose) previously incubated at 37°C for 30 min. Glucose (0.5 M) was injected alone in other group of animals as control (4 treatment groups). Paw volume was measured before s.c. injection of inflammatory stimuli (zero time) and at selected time intervals (1, 2, 3, and 4 h) thereafter by hydroplethysmometry. Calculation was made by subtracting the baseline volume measured at zero time and expressed as the area under the time-course curve in arbitrary units [43].

### Isolation and *In vitro* Stimulation of Neutrophils

Blood from normal volunteers (5 ml) was drawn into 15 ml heparinized (5 IU/ml of heparin) centrifuge tubes. These samples were procured from the blood bank HEMOCE (http:www.hemoce.ce.gov.br). After the lysis of red blood cells with ammonium chloride, neutrophils were isolated by density-gradient centrifugation using Percoll (Sigma). Neutrophils were washed with PBS and resuspended in RPMI 1640 medium supplemented 0.01% of BSA. Neutrophils were incubated with CboL (30, 100 and 300 µg/ml) or with medium alone (negative control) for 1 h at 37°C. After that, neutrophils were centrifuged and washed twice with fresh medium for chemotaxis assay.

### Chemotaxis Assays

Cell migration was assessed by a 48-well microchemotaxis chamber technique as described previously [44]. A 28.6 µL aliquot of chemotaxis stimuli (IL-8 50 ng/mL) was placed in the lower compartment and 50 µL of previously treated cell suspension (1.0×10^6^/ml neutrophils) (as described above) was placed in the upper compartment of the chamber. The two compartments were separated by a polycarbonate filter (5 µm PVP-free polycarbonate filter). The chamber was incubated at 37°C for 1 h. At the end of the incubation period, the filter was removed fixed and stained. The number of migrated cells in five distinct fields was counted by light microscopy after coding the samples. All experiments were repeated at least two times with different cells. The migration was expressed as number of neutrophil per field.

### Statistical Analysis

Results were expressed as mean ± SEM (Standard Error of Mean). For the verification of statistical differences between groups, Analysis of Variance (ANOVA) and the Bonferroni test for multiple comparisons were made. In addition, the Student t-test was used for the rest of the comparisons. Statistical significance was set at *p*<0.05.

## Results and Discussion

### Overall Structure

**Figure 1:**
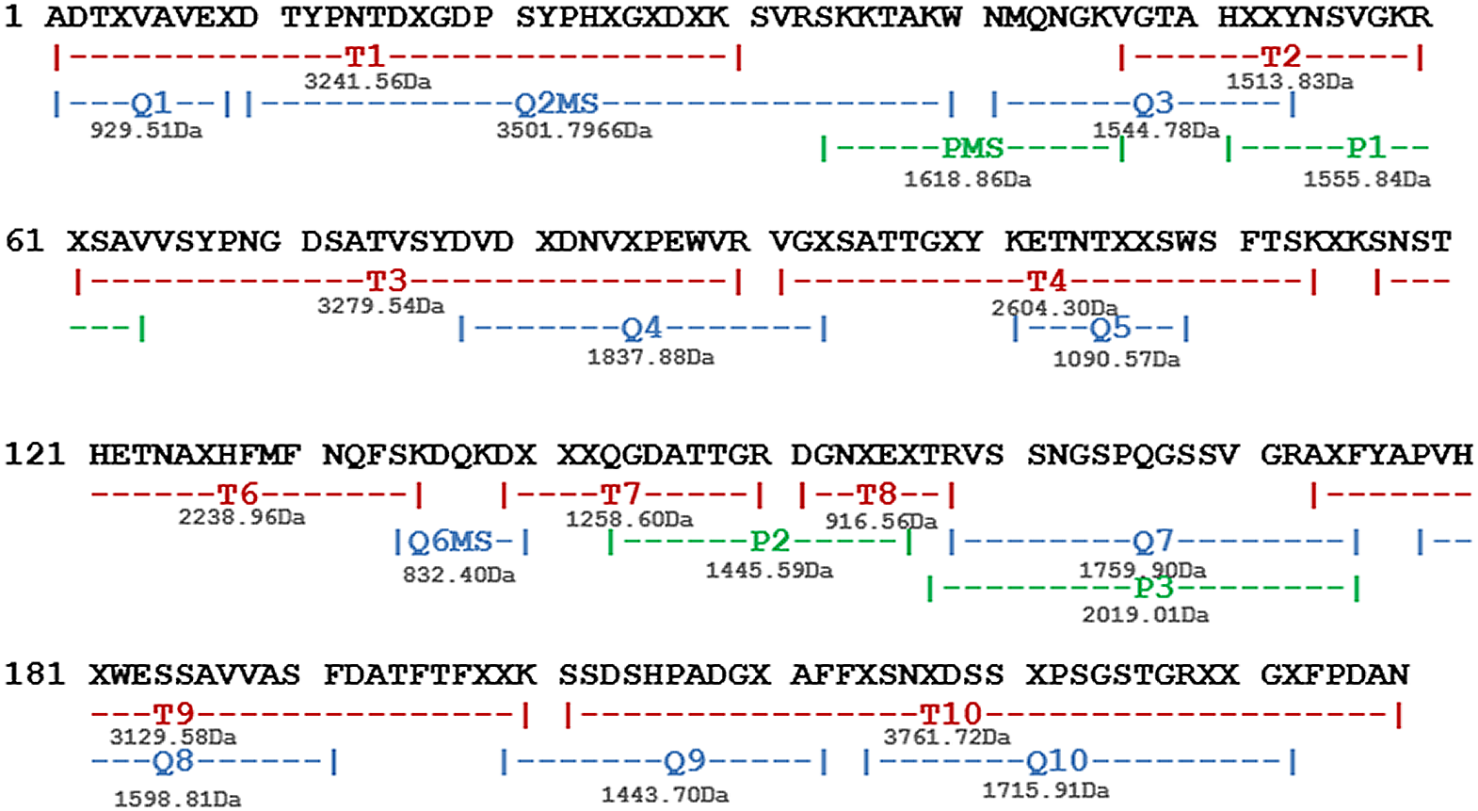
Proposed amino acid sequence of Cbol. Assemble from sequences of degradation products generated by cleavages with trypsin (T-), chymotrypsin (Q-) and pepsin (P-). The letter X in Cbol sequence represents residues of leucine or isoleucine, which cannot be distinguished by mass.

Electrospray ionization mass spectrometry indicated that Cbol consists of a combination of chains weighing 25,572±2 (α-chain), 12,878±1 (β-chain) and 12,710±1 Da (γ-chain). The proposed amino acid sequence of Cbol was established by tandem mass spectrometry analysis (**Figure S1**) using sets of peptides obtained by proteolytic digestions (**Figure 1**). The sequence includes 237 amino acids distributed between the β-chain (residues 1–118) and the γ-chain (residues 119–237). The averaged molecular masses calculated for the full-length α-chain (25,572 Da) and its derived β-(12,878 Da) and γ-(12,710 Da) fragments are in excellent agreement with the experimentally determined masses by ESI-MS. The protein sequence data reported in this paper will appear in the UniProt Knowledgebase under the accession number P86474.

**Figure 2:**
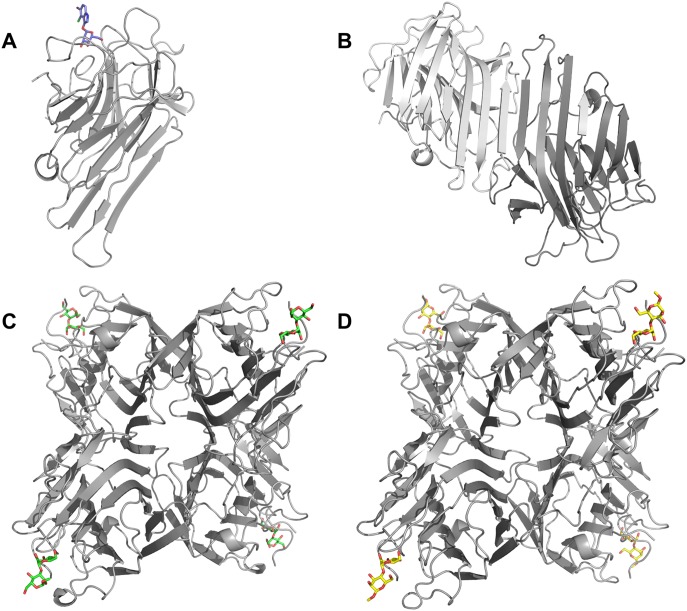
Overall structure of Cbol. Subunits displayed in cartoon representation and colored in grey. (**A**) One molecule in the asymmetric unit of Cbol in complex with Xman (shown as blue sticks). (**B**) Unbound Cbol with two molecules in the asymmetric unit. (**C**) Cbol in complex with M13M (shown as green sticks). (**D**) Cbol in complex with M14M (shown as yellow sticks).

The two structures of Cbol in complex with M13M and M14M exhibit a tetramer formed by two classic ‘‘canonical’’ dimers (subunits A, B, C and D) in the asymmetric unit, while the unbound protein exhibits a single monomer and Cbol:Xman displays a dimer (**Figure 2**), although the Cbol biological oligomer is the tetramer form. As previously observed in other legume lectins, the Cbol monomer consists of 237 amino acids folded as an α/β sandwich [45]. Calcium and manganese binding sites are conserved as previously described for legume lectins [46]. Each metal is coordinated by four residues and two water molecules: Glu8, Asp10, Asp19 and His24 with Mn^2+^, and Asp10, Tyr12, Asn14 and Asp19 with Ca^2+^.

Originating from the crystallization condition, nine cadmium ions are observed in the structures Cbol:M13M and Cbol:M14M. An X-ray fluorescence spectrum measured at DESY (Deutsches Elektronen-Synchrotron), indeed indicated the presence of cadmium ions in the crystal (data not shown). Intriguingly, in all but one subunit of the dimannoside complexes, cadmium ions are found coordinated to two asparagines in the small helix Leu81-Val84 (**Figure S2**).

Other than in the carbohydrate-binding region, the complexes do not present any significant structural differences when compared to the unbound protein. The carbohydrate electron densities of M13M and M14M and Xman complexes are well defined in all subunits. In the dimannoside complexes, none of the subunits are involved in extensive interactions with the symmetry mates around the carbohydrate-binding sites, a favourable feature that prevents bias in the structural analysis of the complexes. In Cbol:Xman, however, extensive interactions are observed in the binding region.

**Figure 3:**
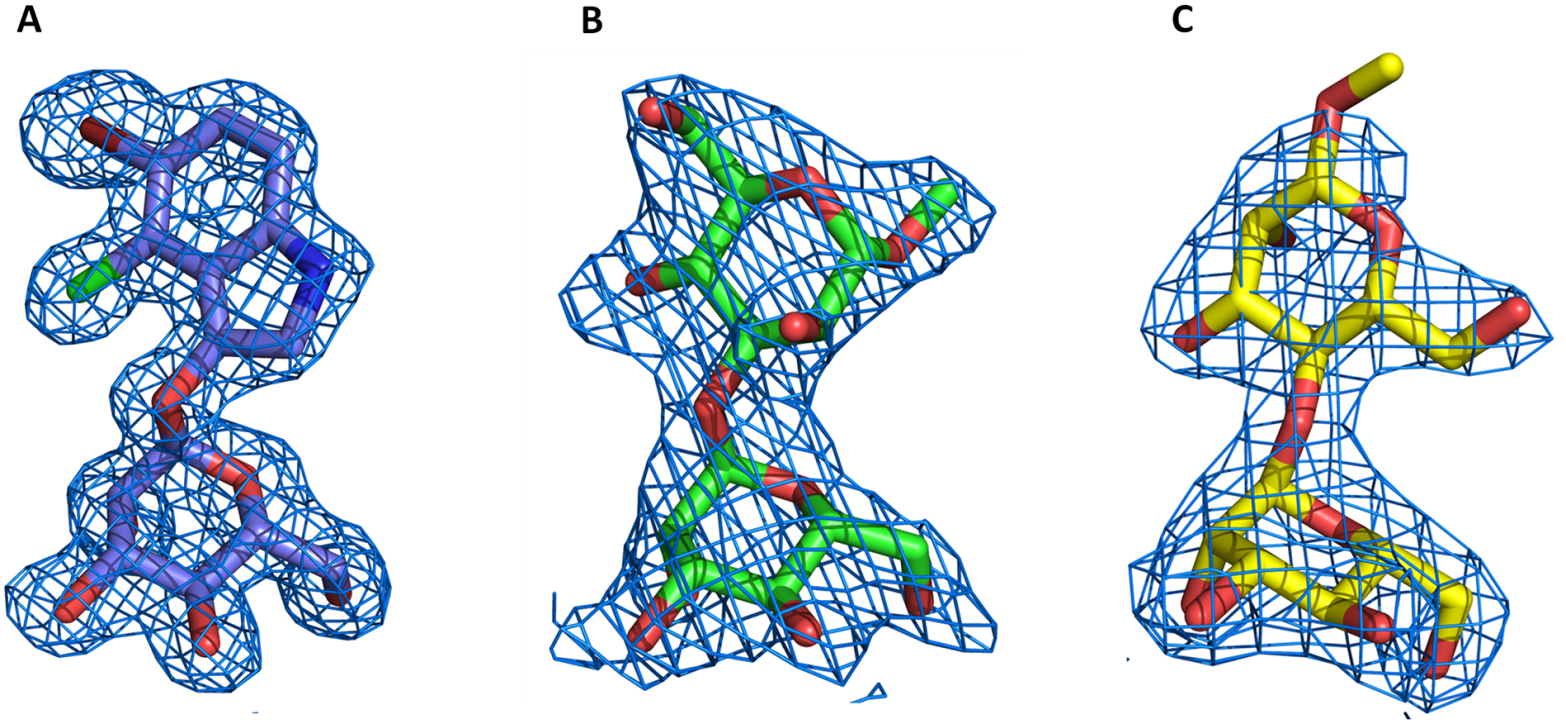
2F_obs_ - F_calc_ electron density maps from the carbohydrates contoured at 1.0 σ. (**A**) Xman. (**B**) Man(α1-3)Man(α1-O)Me, (**C**) Man(α1-4)Man(α1-O)Me.

The electron densities for the three sugars after the final refinement are shown in **Figure 3**. For the M13M and M14M complexes, which crystallized with four molecules in the asymmetric unit, only the most well defined density is displayed. The RMSD between any subunit of all structures described in this study is not higher than 0.3 Å.

### Analysis of Cbol in Complex with M13M, M14M

The O1-linked mannose of both dimannosides, M13M and M14M, as well as Xman, are bound to the monosaccharide binding site in an almost identical way to that observed for ConA, CGL and ConM. The O3- and O4-linked mannose (reducing mannoses, also called here the second mannose) are positioned in a previously described hydrophobic subsite, formed by Tyr12, Leu99 and Tyr100 [19], [47]. The side chains of Tyr12, Leu99 and Tyr100 are opposite to the ring of the reducing mannose, which interacts with the protein mainly by hydrophobic and van der Waals interactions.

**Figure 4:**
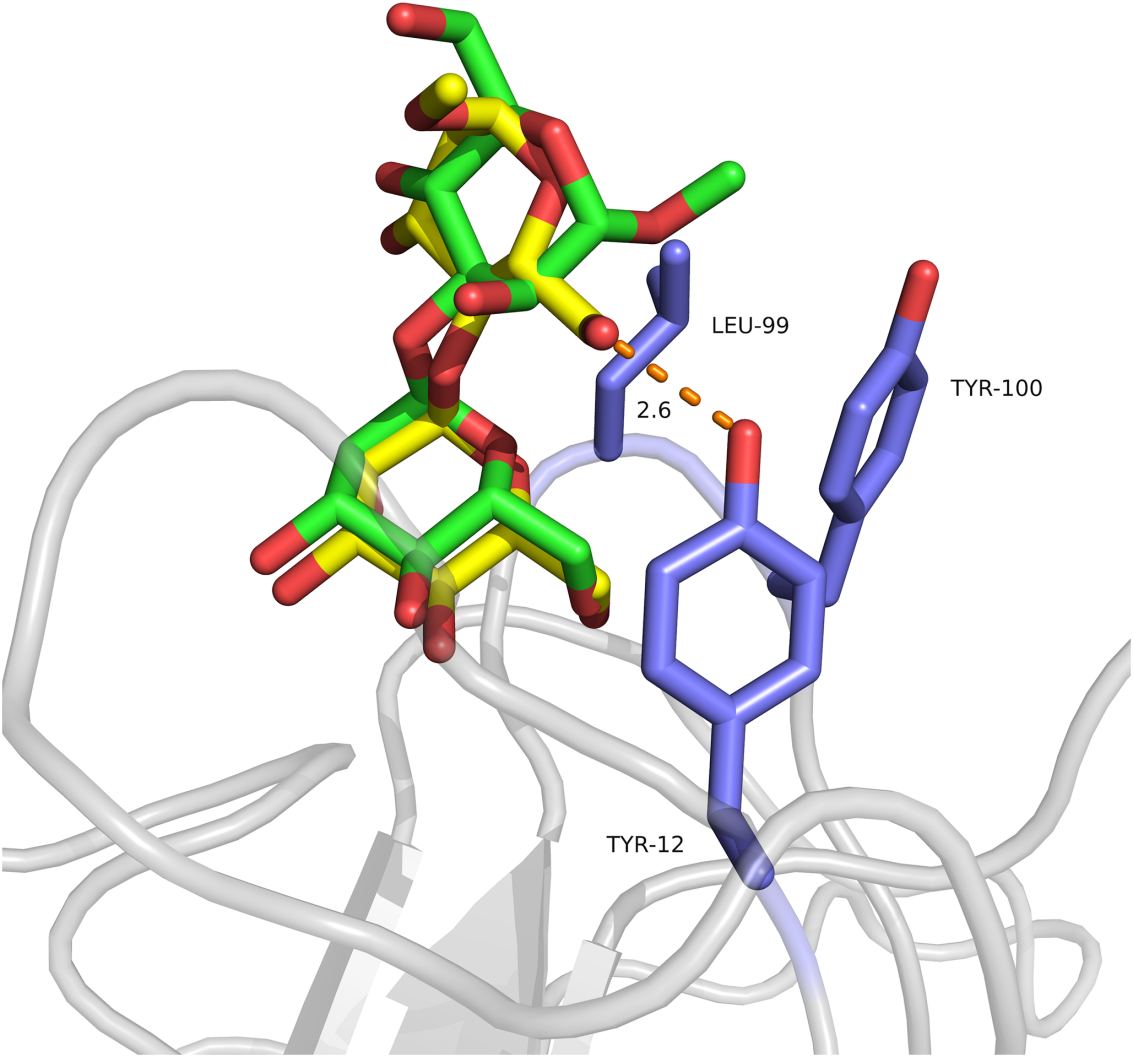
Hydrophobic subsite of Cbol. M14M (shown as yellow sticks) performs one more hydrogen bond with Tyr12 (shown as blue sticks) when compared to M13M (shown as green sticks).

Differences occur in the binding of the dimannosides as result of their distinct glycosidic linkage. When compared to that of M13M, M14M has an additional hydrogen bond formed between the O6 of the reducing mannose and the hydroxyl group of Tyr12 (**Figure 4**). The O6 of M13M is pointing to the opposite direction, away from the hydrophobic subsite.

The extra hydrogen bond established between the hydroxyl group from Tyr12 and the O4-linked mannose might enhance the Cbol affinity for M14M, in comparison to that of M13M. It is important to note that the presence of extra interactions not necessarily result in higher affinity [48]. For instance, ConA:M13M presents a fourfold tighter binding compared to M16M, in spite of less hydrogen bonds and buried surface area [49].

Interestingly, in contrast to Cbol, *Canavalia maritima* lectin and *Canavalia gladiata* lectin display hydrogen bonds between the reducing sugar and the Tyr12 hydroxyl only when in complex with M13M (**Figure S3A**). The reducing mannose of M14M performs only van der Waals interactions with the protein in these cases. Similarly to Cbol, the reducing mannose of M13M when in complex with ConA does not form hydrogen bonds with the protein (**Figure S3B**). However, one cannot exclude that the difference observed in the patterns might be a consequence of the crystal contacts, considering that the carbohydrate-binding sites are in close proximity to symmetry related molecules.

### Structural Basis of the pH-dependent Oligomerization

**Figure 5:**
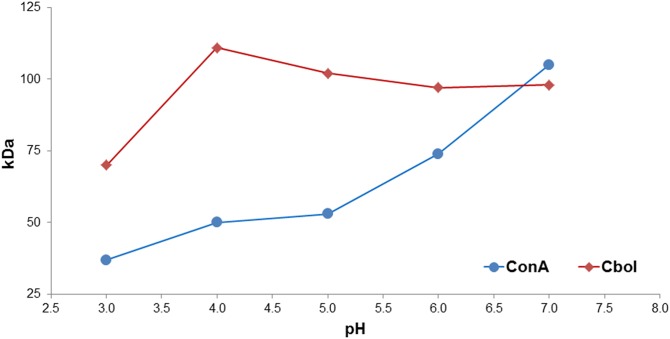
The pH-dependent oligomerization of ConA and Cbol determined with SAXS.

In order to study its oligomerization state, we performed Small Angle X-ray Scattering (SAXS) experiments to determine Cbol molecular masses as a function of the pH (**Figure 5**). For comparison, the same set of measurements was also performed with ConA. Our data indicate that Cbol is mostly in the tetrameric state at pH-values above 4, while ConA, as reported in the literature [50], is predominantly tetrameric only above pH 7.

To study the structural basis of the profiles obtained by SAXS, we analyzed in details the interactions within the tetramer in both protein crystal structures, using the PDBePISA server [51]. The PROPKA web interface [52] was further used to determine the apparent pKa values of residues involved in these interactions (**Table S1 and S2**). For lectin structures with one or two chains in the asymmetric unit, the biological assembly was generated in PyMOL by adding the symmetry mates. This analysis yielded a larger number of polar interactions in the ConA tetramer compared to Cbol. (**Table S3 and Table S4, Figure S4**). On first sight, this observation would suggest that the oligomerization of ConA should be less pH-dependent in clear contradiction to the SAXS data.

**Figure 6:**
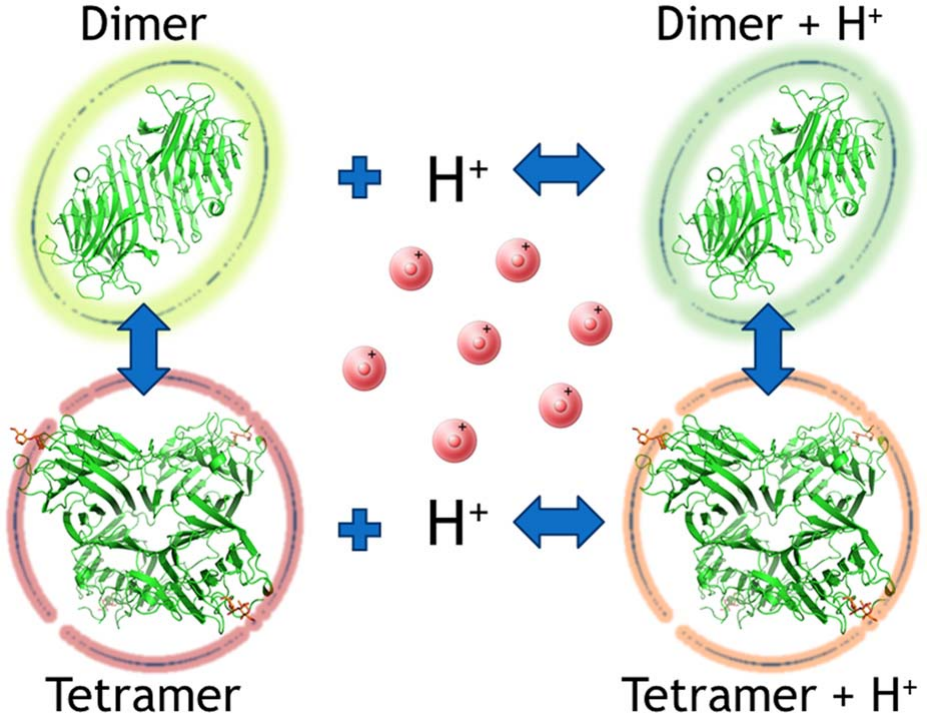
Proposed model for the oligomerization process in Canavalia lectins.

We analyzed the oligomerization behaviour based on the influence of shifted pKa values on protein stability [53], arguing that amino acids with lowered pKa values in the tetramer compared to the dimer will lead to a relative destabilization of the tetramer at lower pH values (**Figure 6**). According to this hypothesis, polar interactions which only involve non-ionizable groups (within biologically reasonable pH ranges) will not influence the relative pH-dependent behaviour. The most prominent effect was observed for Glu192 in ConA with a predicted pKa of 0.3 compared to a pKa of 5.7 for the corresponding Asp192 in Cbol. In the dimer both residues are largely solvent accessible and show only minor pKa perturbations. The large pKa-shift for Glu192 upon tetramer formation in ConA can be explained by salt bridges, which are formed between this residue and Lys114 and Lys116. These ionic interactions are much weaker in Cbol caused by shorter Asp side-chain. Smaller pKa-shift differences were also observed for other residues, such as His51 and His121. Oliveira and coworkers [13] have previously already attributed His51 an important role in the pH-dependent oligomerization of these lectins. They hypothesize that His51 adopts different orientations depending on the environmental pH. It looses its charge at a pH higher than 5, changes the orientation in its neutral state and starts interacting with Lys116 by forming predominately a tetramer.

According to our SAXS data, ConA presents more pronounced pH-dependent dimer-tetramer equilibrium than Cbol. Even though ConA performs more interdimeric interactions, the pKa-shifts of the participating residues very likely induce destabilization at low pH values.

### Unknown Density in the Tetramer Central Cavity

In the central cavity of Cbol in complex with dimannosides, there is a strong unexplained density in the vicinity of His127, Met129 and Asn131 (**Figure S5A**). Although it is possible that this density belongs to some of the components from the crystallization solution, we were not able to interpret it as such.

Curiously, *Dioclea violacea* lectin (DVL) (PDB code: 2GDF) also displays a strong unexplained density in the equivalent area (**Figure S5B**). The components of the crystallization solution differ in both cases: Cbol in complex with dimannosides was crystallized in 0.1 M cadmium chloride hydrate, 0.1 M sodium acetate trihydrate pH 4.6 and 30% (v/v) polyethylene glycol 400, while DVL was crystallized in 10% PEG800 and 10% PEG1000 (source PDB data bank, Gallego del Sol, F. *et al*., 2006). This difference indicates that the observed density is not an artefact of the crystallization experiment.

Since it is well established that lectins can bind to compounds other than carbohydrates [54] we speculate that those densities could originate from a yet to be identified compound present in plant seeds. Since DVL has a Ser129 and His131, while Cbol has a Met129 and Asn131, we wonder if these differences could be an indication of some specificity towards molecules able to bind to the central cavity. Even though highly speculative, we believe that this finding might stimulate further investigation in this direction.

### Biological Activity of Cbol

**Figure 7:**
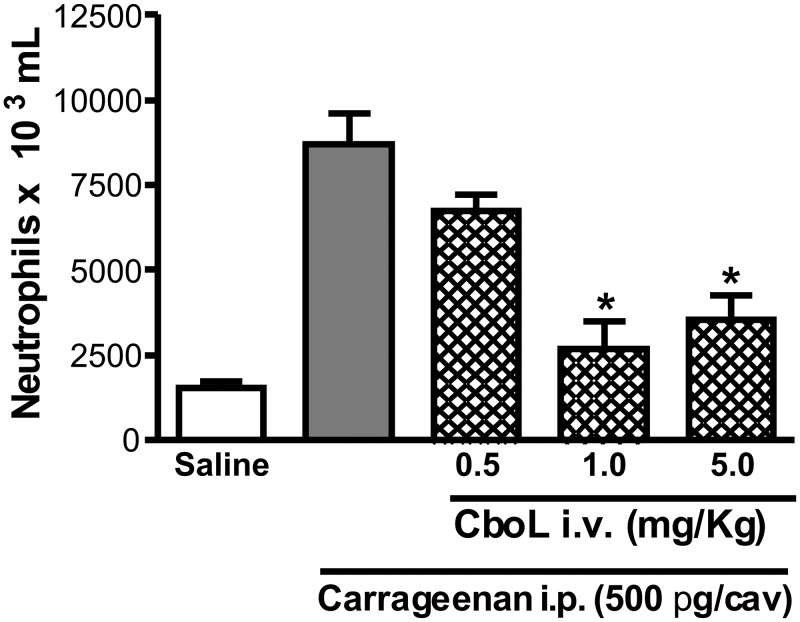
Inhibitory effect of *C. boliviana* lectin (Cbol) upon the neutrophil migration induced by carrageenan. Neutrophil migration was induced by i.p. injection of carrageenan (Cg; 500 µg) and evaluated 4 h later. Animals were treated i.v. (0.1 ml), 30 min before stimuli with: Saline and CboL (0.5, 1 and 5 mg/Kg). Values represent mean ± S.E.M. (n = 6); *indicate significant differences from Cg alone. (p<0.05) ANOVA-Bonferroni.

**Figure 8:**
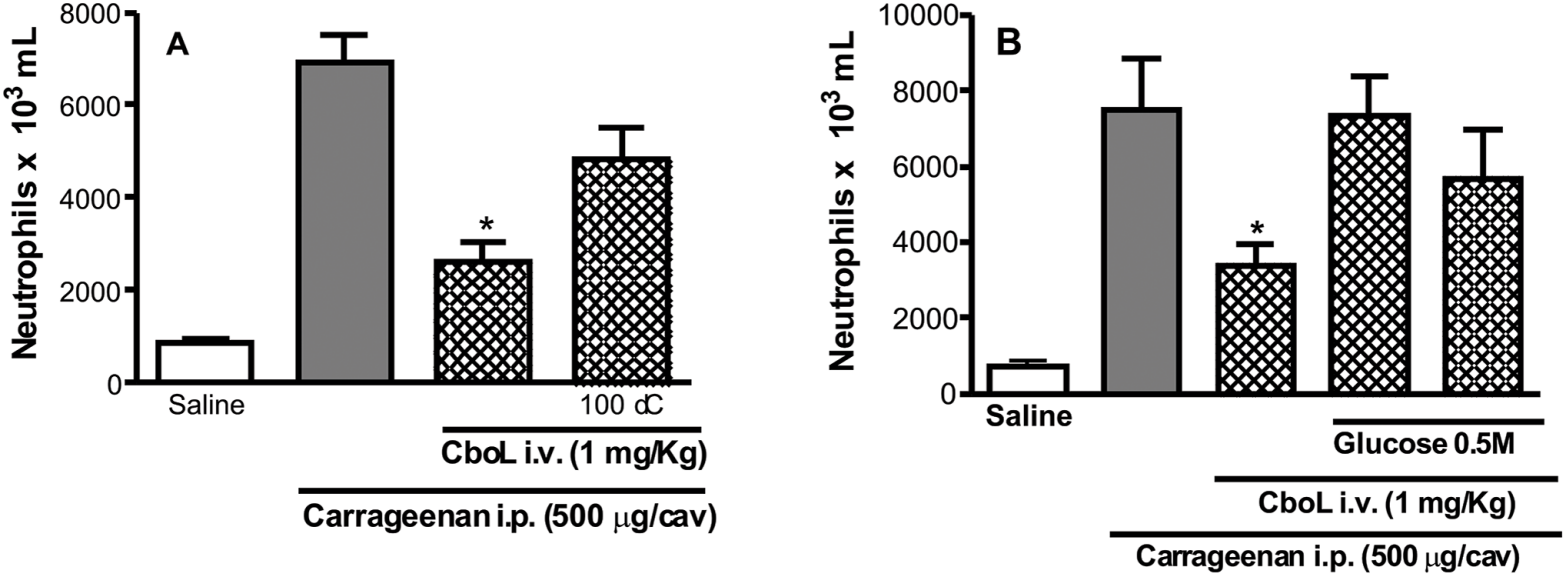
Inhibitory effect of carbohydrate and heat-treated on anti-inflamatory effect induced by Cbol. (**A**) CboL (1 mg/Kg i.v), native or heat-treated (100°C) for 30 min; (**B**) CboL (1 mg/Kg i.v) alone or combined with α-methyl-D-Glucose (0.5 M). The black bar represents neutrophil migration induced by the injection of the carbohydrate alone. Values are reported as means ± S.E.M. for six animals. *p<0.05 compared with carrageenan (Cg) alone (analysis of variance-Bonferroni).

The neutrophil migration induced by i.p. injection of carrageenan was inhibited by Cbol at concentrations of 1 and 5 mg/Kg (**Figure 7**), indicating its anti-inflammatory effect. This effect was reverted when the protein was previously thermally denatured at 100°C (**Figure 8A**) or incubated with α-methyl-mannoside at 0.5 M (**Figure 8B**), the latter, indicating that the activity is related to the lectin carbohydrate recognition domain (CRD). On the other hand, the pro-inflammatory effect of neutrophil migration to peritoneal cavity after i.p. administration of lectins was systematically reported to many Diocleinae lectins such as *Dioclea rostrata* lectin (DRL) [55].

**Figure 9:**
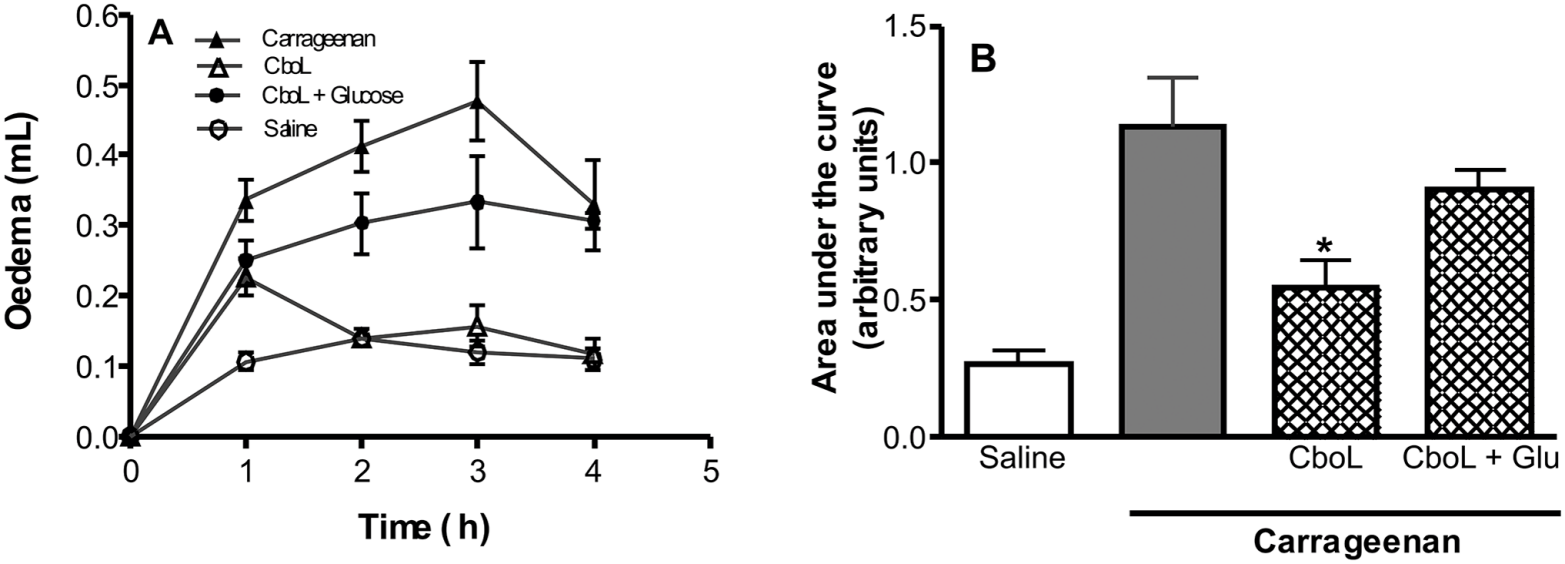
Inhibitory effect of *C. boliviana* lectin (Cbol) upon the rat paw oedema induced by carrageenan: reversion by glucose. Paw oedema was induced by s.c. injection of carrageenan (Cg; 500 µg). Animals were treated i.v. (0.1 ml), 30 min before stimuli, with: Saline or CboL (1 mg/Kg; i.v) alone or associated to glucose (0.5 M). Control groups received saline i.v. and Cg s.c. or only saline s.c. (**A**) Oedema was measured at 1, 2, 3 and 4 h after stimuli and expressed as the increase in paw volume (ml). (**B**) The area under the time-course curve was determined using a trapezoidal rule. Values represent mean ± S.E.M. (n = 6). *indicate significant differences from Cg alone (p<0.05) ANOVA-Bonferroni.

The paw oedema induced by carrageenan was also inhibited in presence of Cbol at 1 mg/Kg (**Figure 9**). This effect was also reverted when the protein was incubated with 0.5 M glucose. Some Phaseoleae lectins, isolated from *Canavalia grandiflora*, *C. ensiformis* [56], *Dioclea rostrata* [*55*], *C. brasiliensis*, *C. gladiata*, *C. maritima* (Assreuy *et al*., 2009) and *Cymbosema roseum* [57], elicit inflammatory responses that could be reversed by specific carbohydrates. *Cymbosema roseum* is also known to elicit anti-inflammatory responses after systemic administration, which has been described as a legume lectin pattern of action; an inflammatory response based on administration route [57]. Cbol anti-inflammatory action is very consistent because describes anti-inflammatory responses through the i.v. administration route, in accordance with this pattern.

The oedema increased in the first hour and a partial inhibition was observed, the next three hours revealed an oedema reduction until levels similar to the control in the fourth hour. In the paw oedema model, ConBr, CGL, ConM and CRLI show acute oedematogenic activity. Among them, ConM is the different one in oedema induction without NO participation, lower antinociceptive efficacy or higher efficacy and potency in vasorelaxant action. Similar to ConM, *Canavalia grandiflora* lectin acts as anti-inflammatory and analgesic [58] and has an oedematogenic activity in rat paw oedema model (s.c.), but has a vasorelaxant effect in endothelized rat aorta [59].

**Figure 10:**
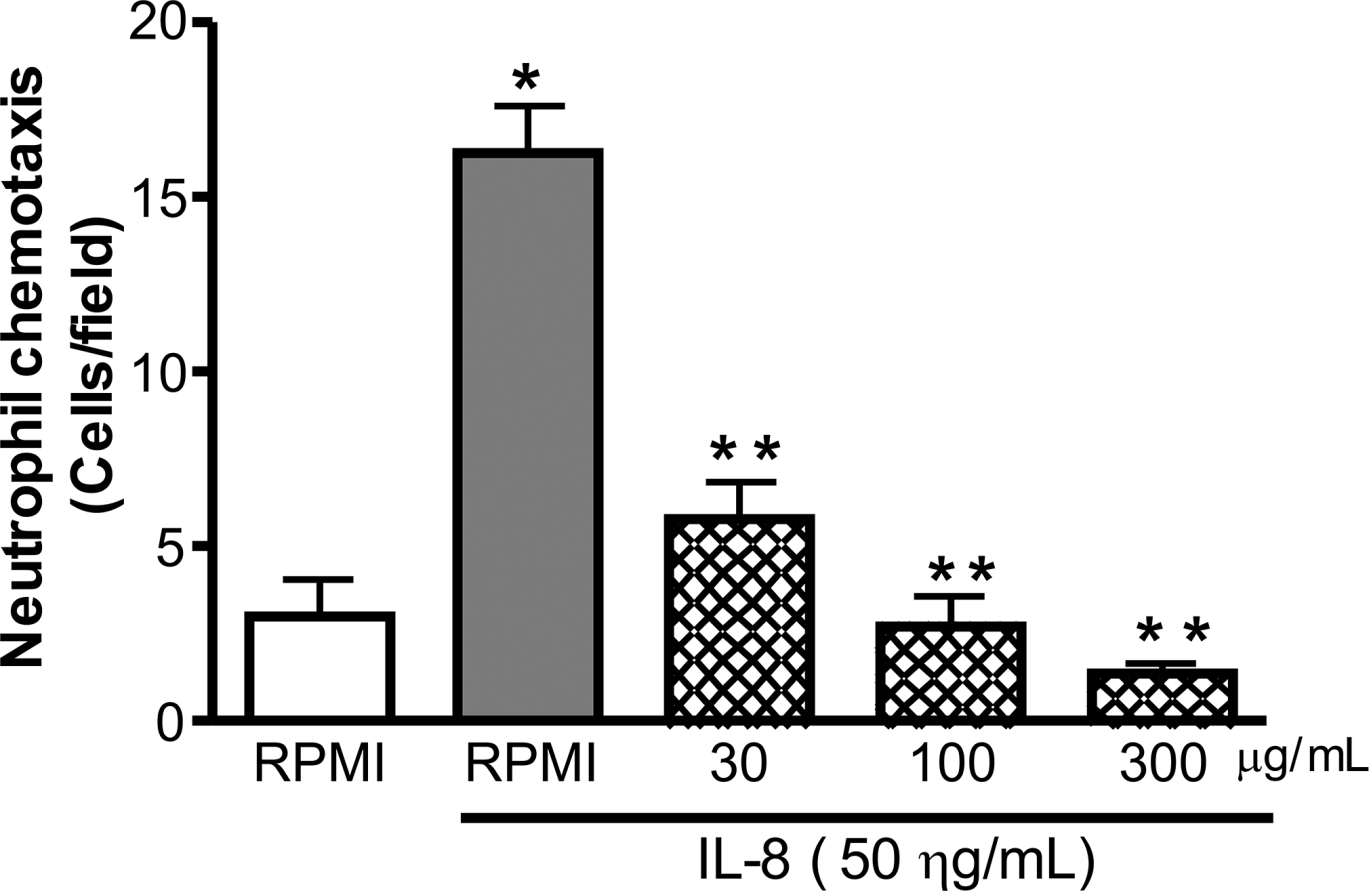
Chemotaxis assay of human neutrophils incubated in the presence of Cbol. Cells were allowed to migrate in the Boyden chamber toward the medium alone or IL-8. Cells were pre incubated with different concentrations of CboL. Data show means ± SD from three independent experiments performed in triplicate.

In animal models, carrageenan stimulates resident cells to release chemotactic inflammatory mediators, increasing leukocyte migration, especially neutrophils [60]. On the other hand, fMLP acts directly as a chemo attractant on leukocytes [61]. The chemotaxis stimuli induced by IL-8 was significantly reverted by all the concentrations tested at levels below 5 neutrophil cells per field, being very significant compared to positive control (RMPI buffer + IL8) (**Figure 10**). This is an indicative that Cbol acts on resident cells, inhibiting the release of pro-inflammatory cytokines and/or stimulating the release of anti-inflammatory cytokines.

Lectins can display pro or anti-inflammatory activities depending on the administration route. Systemically, *Dioclea violacea*, *D.guianensis*, *D.virgata* e *Cratylia floribunda* lectins inhibit neutrophil migration to peritonial cavity induced by carrageenan or fMLP and rat paw oedema also induced by carrageenan [7]; [62], events that regulates directly or indirectly the neutrophil migration.

## Conclusion

Our structural analyses of the Cbol provide a rationale for the differences observed in the biological activities of lectins belonging to the *Diocleinae* subtribe. Particularly, our model for the pH-dependent oligomerization sheds some light into the dimer-tetramer equilibrium and its importance for the distinct activities observed in this class of proteins. Furthermore, we characterized the anti-inflammatory properties of Cbol, adding more data to the repertoire of wide biological activities displayed by this group of lectins, despite their highly identical sequence.

## Supporting Information

Figure S1 **Collision-induced dissociation of the triply charged ion at** ***m/z*** **1255.25 corresponding to the T10 peptide of Cbol.** The sequence-specific y- and b- ions used for the sequence determination are indicated.
(TIF)

Figure S2 **Interaction of Cadmium ion with Asp82 in the Helix Leu81-Val84 and the residue Asp80, in Cbol:M13M, chain B.**
(TIF)

Figure S3 **Dimannoside M13M interactions.** (**A**) Interaction with ConM (PDB code 2P37). Two hydrogen bonds are formed with the hydroxyl group of Tyr12. The same pattern is also observed for CGL. (**B**) Interaction with ConA (PDB code 1QDO), similarly to Cbol, the reducing mannose does not perform any hydrogen bond with the protein.
(TIF)

Figure S4 **Hydrogen bond interactions.** (**A**) Dimeric interface of Cbol:Xman and ConA (PDB code 1NLS). (**B**) Tetrameric interface of Cbol:Xman and ConA (PDB code 1NLS).
(TIF)

Figure S5 **Unexplained (Fo-Fc) electron density.** (**A**) Central cavity of Cbol M13M displayed at 2.5 σ, markedly interacting with residues His127, Met129 and Asn131; and (**B**) *Dioclea violaceae* lectin displayed at 3.0 σ (PDB code: 2GDF), markedly interacting with His 127, Ser129 and His131.
(TIF)

Table S1 **Comparison of the dimeric and tetrameric pKa values of Cbol.** Significantly lower pKa values are marked in bold.
(TIF)

Table S2 **Comparison of the dimeric and tetrameric pKa values of ConA.** Significantly lower pKa values are marked in bold.
(TIF)

Table S3 **Hydrogen bonds and salt bridges within the tetrameric interface of ConA.**
(TIF)

Table S4 **Hydrogen bonds and salt bridges within the tetrameric interface of Cbol.**
(TIF)

Arrive Checklist S1 **Report of the animal experiments.**
(PDF)
