# Supplementary figures and images for: Structural Studies of an Anti-Inflammatory Lectin from Canavalia boliviana Seeds in Complex with Dimannosides
Source: PLoS One. 2014 May 27;9(5):e97015. doi: 10.1371/journal.pone.0097015 (PMC4035259; doi:10.1371/journal.pone.0097015)

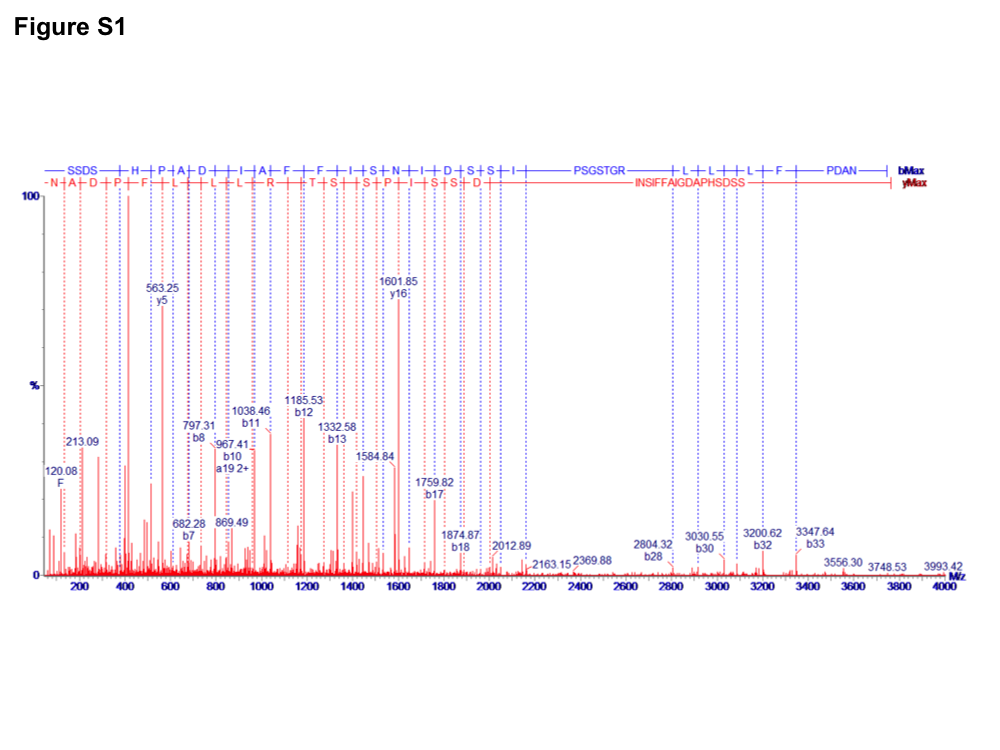

Supplement: Figure S1 — Collision-induced dissociation of the triply charged ion at m/z 1255.25 corresponding to the T10 peptide of Cbol. The sequence-specific y- and b- ions used for the sequence determination are indicated. (TIF) [file pone.0097015.s001.tif]

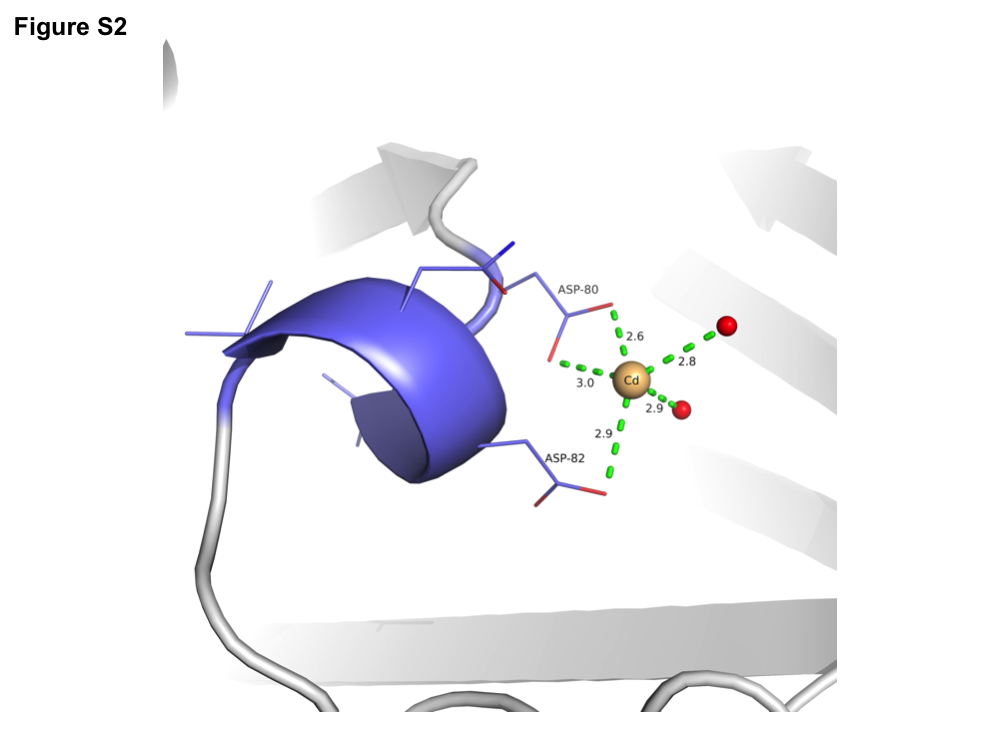

Supplement: Figure S2 — Interaction of Cadmium ion with Asp82 in the Helix Leu81-Val84 and the residue Asp80, in Cbol:M13M, chain B. (TIF) [file pone.0097015.s002.tif]

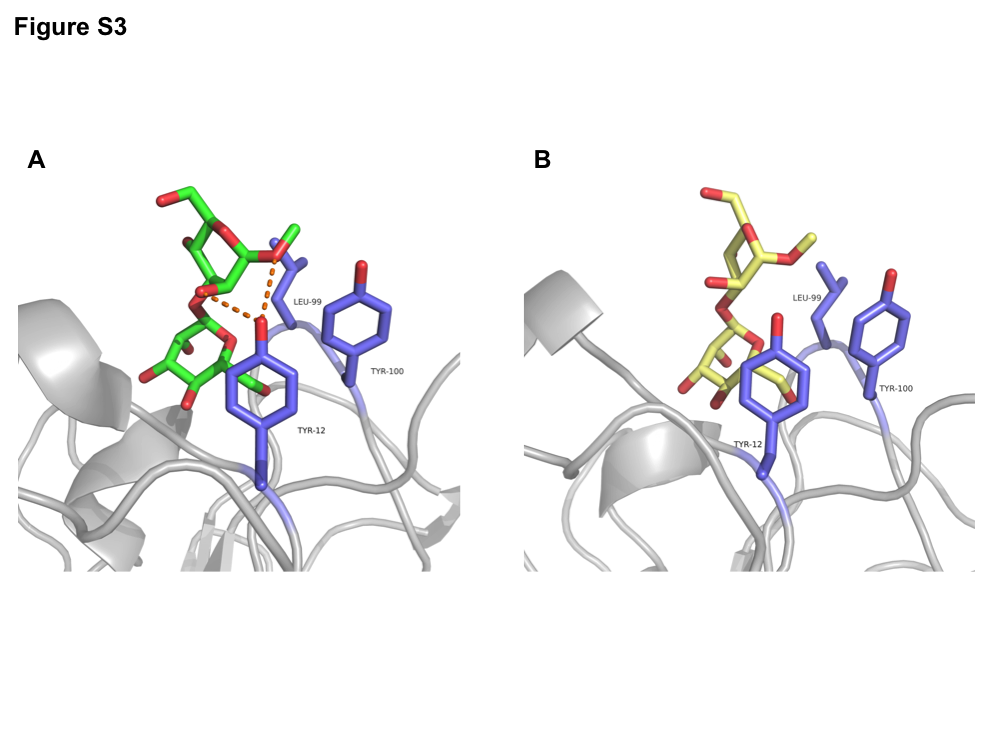

Supplement: Figure S3 — Dimannoside M13M interactions. (A) Interaction with ConM (PDB code 2P37). Two hydrogen bonds are formed with the hydroxyl group of Tyr12. The same pattern is also observed for CGL. (B) Interaction with ConA (PDB code 1QDO), similarly to Cbol, the reducing mannose does not perform any hydrogen bond with the protein. (TIF) [file pone.0097015.s003.tif]

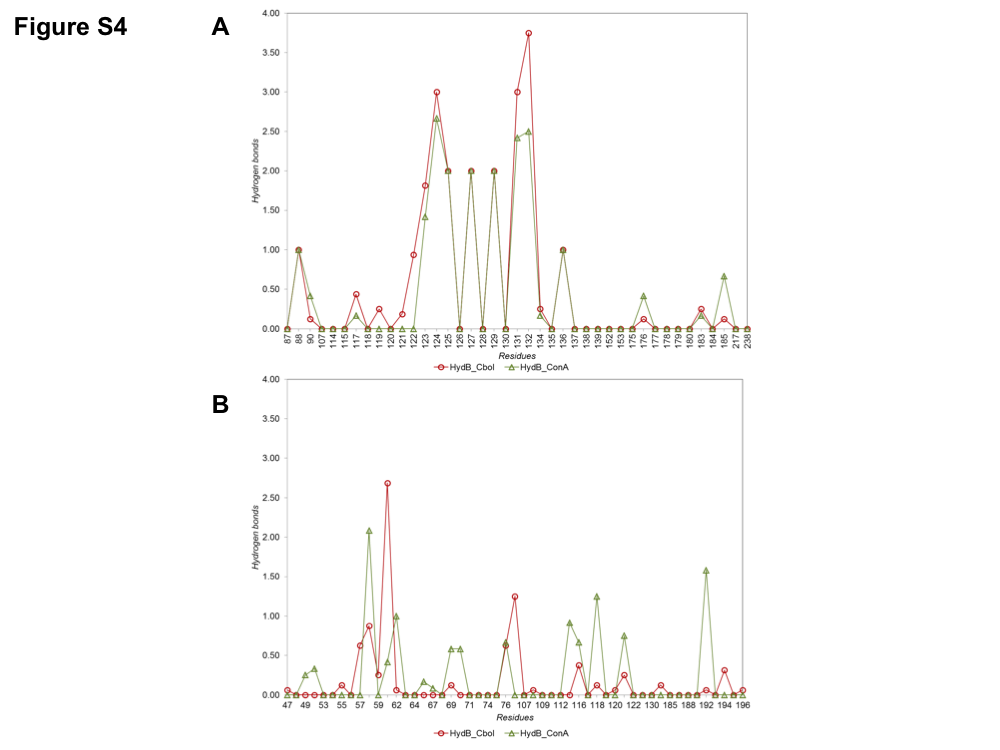

Supplement: Figure S4 — Hydrogen bond interactions. (A) Dimeric interface of Cbol:Xman and ConA (PDB code 1NLS). (B) Tetrameric interface of Cbol:Xman and ConA (PDB code 1NLS). (TIF) [file pone.0097015.s004.tif]

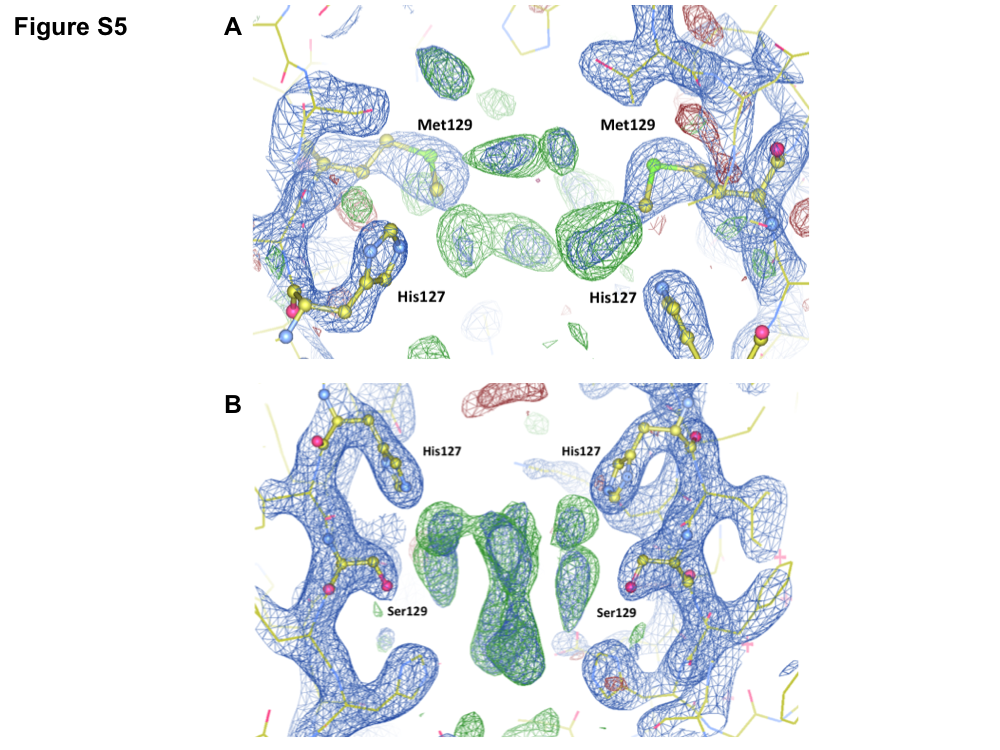

Supplement: Figure S5 — Unexplained (Fo-Fc) electron density. (A) Central cavity of Cbol M13M displayed at 2.5 σ, markedly interacting with residues His127, Met129 and Asn131; and (B) Dioclea violaceae lectin displayed at 3.0 σ (PDB code: 2GDF), markedly interacting with His 127, Ser129 and His131. (TIF) [file pone.0097015.s005.tif]
